# Challenges and lessons from a school-based human papillomavirus (HPV) vaccination program for adolescent girls in a rural Nigerian community

**DOI:** 10.1186/s12889-022-13975-3

**Published:** 2022-08-24

**Authors:** Michael Egbon, Tolulope Ojo, Aminu Aliyu, Zainab Shinkafi Bagudu

**Affiliations:** 1 Medicaid Cancer Foundation, Abuja, Nigeria; 2 Institute of Health Policy, Management and Evaluation, University of Toronto, Toronto, Canada

**Keywords:** Cervical cancer, Vaccination, Human papillomavirus (HPV), Adolescent girls, Nigeria

## Abstract

**Background:**

Over 80% of new cervical cancer cases occur in women living in low- and middle-income countries. It is the second highest cause of female cancer deaths in Nigeria. School based vaccination programs are an effective strategy for delivering the HPV vaccine to adolescent girls. This study aims to understand the challenges to implementing school-based HPV vaccination programs, particularly in a remote rural setting where vaccine hesitancy is high.

**Methods:**

A 22- item interviewer administered questionnaire was used to evaluate HPV knowledge and willingness to get the HPV vaccinate among 100 female secondary school students as part of an HPV vaccination pilot in a rural community in Kebbi State, Nigeria. Additionally, semi-structured interviews were used to assess community knowledge and attitudes on cervical cancer and HPV vaccination. Data collected were analyzed thematically to understand challenges and generate lessons for vaccine delivery in the study setting.

**Results:**

Knowledge of HPV and cervical cancer among junior secondary school aged girls was fair with a mean score of 66.05%. For senior secondary school aged girls, the knowledge score ranged from 70 to 100% with a mean of 96.25% indicating good knowledge of HPV and cervical cancer. All participants (*n* = 100) received the first vaccine dose but due to COVID-19, 33 participants were not able to complete the vaccine dosage within the recommended 6-month schedule. Of the parents who provided consent, none could afford the vaccine out of pocket. Challenges to vaccine delivery included operational costs exacerbated by lack of adequate health workforce and infrastructure in the study setting.

**Conclusion:**

An exploration of sociocultural perspectives and contextual realities is crucial to understanding the complexities of HPV vaccine introduction from the perspective of the target audience, and the local community. Strategies for introducing the HPV vaccine should address community concerns through effective communication, appropriate delivery, and targeted advocacy to make the vaccination program locally relevant. While school-based HPV immunization programs have been shown to be successful, adequate design, planning and monitoring is important. Additionally, considerations must be made to account for the high operational cost of vaccine delivery in rural, hard to reach areas where human resources and infrastructure are limited.

## Background

Cervical cancer is a major public health issue globally with over 600,000 new cases and 300,000 deaths in 2020 [1]. Over 80% of incidence and mortality is in low-and-middle income countries (LMICs) with the highest mortality in sub-Saharan Africa [1, 2]. Infection with the high risk human papillomavirus (HPV) is primarily responsible for cervical cancer [3]. HPV is sexually transmitted, and most people become infected sometime during their lifetime, usually soon after becoming sexually active. In Nigeria, cervical cancer accounts for the second highest cancer burden among women of all ages, with 12,075 new cases and 7968 deaths in 2020 [4].

The World Health Organization (WHO) estimates that at least one third of all HPV-related cancers in Africa could be prevented with vaccination [5, 6]. Specifically, HPV vaccination for girls aged 9–14- years old is recommended to prevent HPV infection [6, 7]. Consequently, universal vaccination of adolescent girls is now part of the routine immunization schedule in many high income countries [6, 8]. Five of the countries with the highest numbers of deaths from cervical cancer are in Sub-Saharan Africa, highlighting the need for increased uptake of HPV vaccination in the region [5]. As such, it is imperative that countries implement HPV delivery strategies that are effective, affordable, sustainable, and compatible with their health systems to achieve the highest possible coverage. Since 2012, several global and national programs have introduced pilot programs to determine the best strategies to optimize HPV vaccine delivery in LMICs [9–11].

There are currently three licensed prophylactic HPV vaccines, two of which are available to LMICs. Cervarix®, a bivalent vaccine that targets HPV 16 and 18, and, Gardasil®, a quadrivalent vaccine which additionally targets HPV 6 and 11 that cause genital warts [3]. Delivery of HPV vaccination at secondary schools provides an ideal route to reaching the target population [2]. In Nigeria, secondary school enrollment increased from 64% in 2010 to 78% in 2019, and presents an ideal route for reaching Nigerian girls aged between 9 and 14 [12, 13]. However, while there have been plans to expand the national routine immunization schedule to include HPV vaccine, several factors including global supply constraints and sociocultural barriers including the mistrust of western medicine remain challenges [14, 15]. Therefore, as Nigeria prepares to introduce HPV vaccination into its national routine immunization schedule, evidence-based research to identify effective delivery strategies is needed to optimize vaccination uptake. In line with the WHO guidelines, the Nigerian Cervical Cancer Control Strategy recommends school- based delivery of HPV vaccine. Roughly 1 in 10 adolescents in Nigeria is sexually active and, historically, rural settings have disproportionately high levels of vaccine hesitancy [2, 16]. Understanding the unique contextual factors that impact HPV vaccine delivery in a rural setting is a crucial step towards the design of effective, targeted vaccine roll out strategies.

As such, the aim of this study was to investigate results from a pilot vaccination program targeting adolescent girls in Yauri, Kebbi state – a rural community in Northern Nigeria. The study examines contextual factors and challenges to implementation of vaccine delivery in a low-resource, rural setting.

## Methods

### Description of HPV vaccination program

This study was conducted as part of a broader program of activities to improve cervical cancer awareness, screening, and treatment under the Kebbi State Strategic Plan for Cancer Control (2019–2023). In line with the Nigerian National Cancer Control Plan which recommends school-based vaccination of secondary school aged girls, Medicaid Cancer Foundation (MCF), in collaboration with the Kebbi State Ministry of Health and Jaiz Charity and Development Foundation (JCDF) piloted an HPV vaccination program targeting adolescent girls in Yauri, Kebbi state – a rural community in Northern Nigeria. Cervarix® was selected as the vaccine of choice as it guarantees nearly 100% protection against HPV type 16 and 18, the two most common strains of HPV in Nigeria and has limited side effects [17].

### Community mobilization and advocacy

To prepare for the pilot vaccination program, some time was spent sensitizing the Emir of Yauri, who is the traditional leader of the community to explain the purpose of the study. This was followed by a day of community sensitization exercises to educate the community on cervical cancer. As part of the sensitization campaign, meetings were organized with the community members, the Parent Teacher’s Association, school staff as well as representatives from the ministry of education.

### Study design and participants

The study was a descriptive cross-sectional study conducted to assess knowledge, attitudes and perceptions on HPV, the HPV vaccine and cervical cancer. The vaccination program was rolled out at Girls Science Secondary College (GSSC) Yauri, Kebbi state between February and July 2020. The school is in Yauri Town, a remote community in the Yauri Local Government Area of Kebbi State, an area that covers 315.1 km^2^ with a population of 100,564 people [18]. The school is located about 180 km away from the state capital, Birnin-Kebbi, Northwest Nigeria. GSSC Yauri is a girls-only boarding school comprising of Junior and Senior Secondary School girls aged between 9 to 19 years old. The study site was purposively selected as a low resource setting with no previous history HPV vaccination delivery to allow for administration of the complete Cervarix® dosage. Intramuscular injection of Cervarix® HPV vaccine was delivered based on the standard dosing schedule by trained nurses (Table 1).

**Table 1:** Cervarix® dosing schedule

Cervarix® Dosing Schedule		
Age at first injection	Immunization and Schedule	Flexibility of immunization if required
From 9 years up to and including 14 years	2 dose each of 0.5 ml at 0 and 6 month	Second dose between 5 and 13 month after first dose
From 15 years and above	3 dose each of 0.5 ml at 0, 1 and 6th month	Second dose between 1 and 2.5 months after 1st dose. Third dose between 5 and 12 month after first dose

The final number of participants selected for the study was based on the funding provided and cost of vaccine delivery. A total of 100 participants (~ 11% of the total school’s population) between the ages of 9 to 18 years were randomly selected for the study. Based on a 95% confidence interval and a power of 80%, this sample size provides a 9% margin of error. Girls below 9 and above 18 years of age, those who were sexually active, or whose parent or guardian did not consent to HPV vaccination were excluded from the program.

### Data collection and analysis

The instrument used for data collection was a pre-tested 22- item interviewer administered questionnaire developed through a literature search of previous studies [19–22]. The questionnaire was used to collect information about sociodemographic characteristics (sex, age, religion, class year, etc.) and assess participant knowledge. Seven questions were used to assess the study participants’ knowledge on cervical cancer and HPV vaccines. The response options were either dichotomous (Yes/ No, or True/False) or multiple choice. Every correct response was given a score of ‘1’ and other responses were given a score of ‘0’. The total knowledge score was calculated for each participant, with the highest score being 100. A score below 50 represents “poor” knowledge, scores between 50 and 80 are fair, and scores above 80 indicate good knowledge of HPV and cervical cancer. Questions related to affordability and acceptability of the vaccines were also posed to the students. Questions specific to the school setting—such as logistics, involvement of school staff, disruptions, and coordination with the health and education ministry—were posed to the school management. Furthermore, relevant stakeholders including community health workers, school staff and the community traditional leaders were purposively selected for interviews to provide wider insights on potential contextual barriers and facilitators of HPV vaccination delivery in the study setting.

To ensure privacy interviews were conducted in a classroom, where only one girl was interviewed at a time by two health workers. All data were collected and stored on a secure computer database accessible only by Medicaid Cancer Foundation (MCF) staff and researchers. Descriptive statistics were generated, and data presented as means (standard deviation) for continuous variables and as percentages for categorical variables.

### Consent

Verbal consent was solicited from parents in the local Hausa language via phone using a pre-defined script due to logistics and language limitations. Parents providing consent were noted and the school vice principal signed the girls consent forms, serving as the parents authorized representative. In addition to obtaining consent from parents, the method of verbal consent was reviewed and approved by the school management and members of the Parents Teacher Association Board (PTA).

### Ethics

The study protocol was reviewed and approved by the Health Research Ethics Committee of the Kebbi State Ministry of Health, Nigeria (reference number: 106: 44/2021).

## Results

### Participant characteristics

Table 2 shows the characteristics of the study participants (*n* = 100). Study participants received their first dose of Cervarix® on February 20, 2020. For age, data are expressed as mean values with their standard deviation (SD). The mean age of the vaccinated students was 13.32 years with a SD of 3.1 years. Seventy participants were enrolled in junior secondary school while 30 were enrolled in senior secondary school.

**Table 2:** Characteristics of study participants. (*n* = 100)

Variable	Proportion (%)	Mean (+) SD
**Age (years)**		13.32 + 3.1
Early adolescence (9–14 years)	70	13.2 + 1.3
Late adolescence (14–18 years)	30	15.7+ 1.7
**Class**		
^a^JSS 1	26	
JSS 2	19	
JSS3	31	
^b^SSS 1	4	
SSS 2	20	
SSS 3	–	
**Religion**		
Muslim	87.3	
Christian	19.3	

^a^*JSS* Junior Secondary School

^b^*SSS* Senior Secondary School

### Knowledge on HPV infection and cervical cancer

Most students had some knowledge of cervical cancer with students scoring an average of 73.3% on the administered questionnaire. However, while 81.3% of the students correctly linked HPV to any cancer only 52% correctly linked HPV to cervical cancer specifically. Additionally, only 58.7% correctly responded that HPV was sexually transmitted and 32.2% of the students were not aware that the HPV vaccine could prevent cervical cancer (Table 3). Overall, student knowledge on HPV improved with class year; junior secondary students scored lower compared to their senior secondary counterparts.

**Table 3:** Student HPV knowledge on HPV and Cervical cancer

**Questions**	**Yes (%)**	**No (%)**	
Knows what Human papillomavirus (HPV) is	86.7	17.3	
Knows the type of cancer caused by HPV	52	48	
Understands why girls need to be vaccinated with the HPV vaccine	64	36	
Knows HPV is primarily transmitted through sexual intercourse	58.6	41.4	
Understands who can be infected with HPV (sex)	24	76	
Knows HPV may be linked to any cancer	81.3	18.7	
Understands purpose of pap smear	73.3	26.7	
**Overall knowledge score**^**a**^	**Poor**	**Fair**	**Good**
Junior secondary school (*n* = 76)	24	29	23
Senior secondary school (*n* = 24)	–	3	21

^a^Overall Knowledge Score: < 50% = Poor knowledge; 50–80% = Fair Knowledge; 80 0% and above = Good Knowledge

### Vaccination coverage

**Fig. 1:**
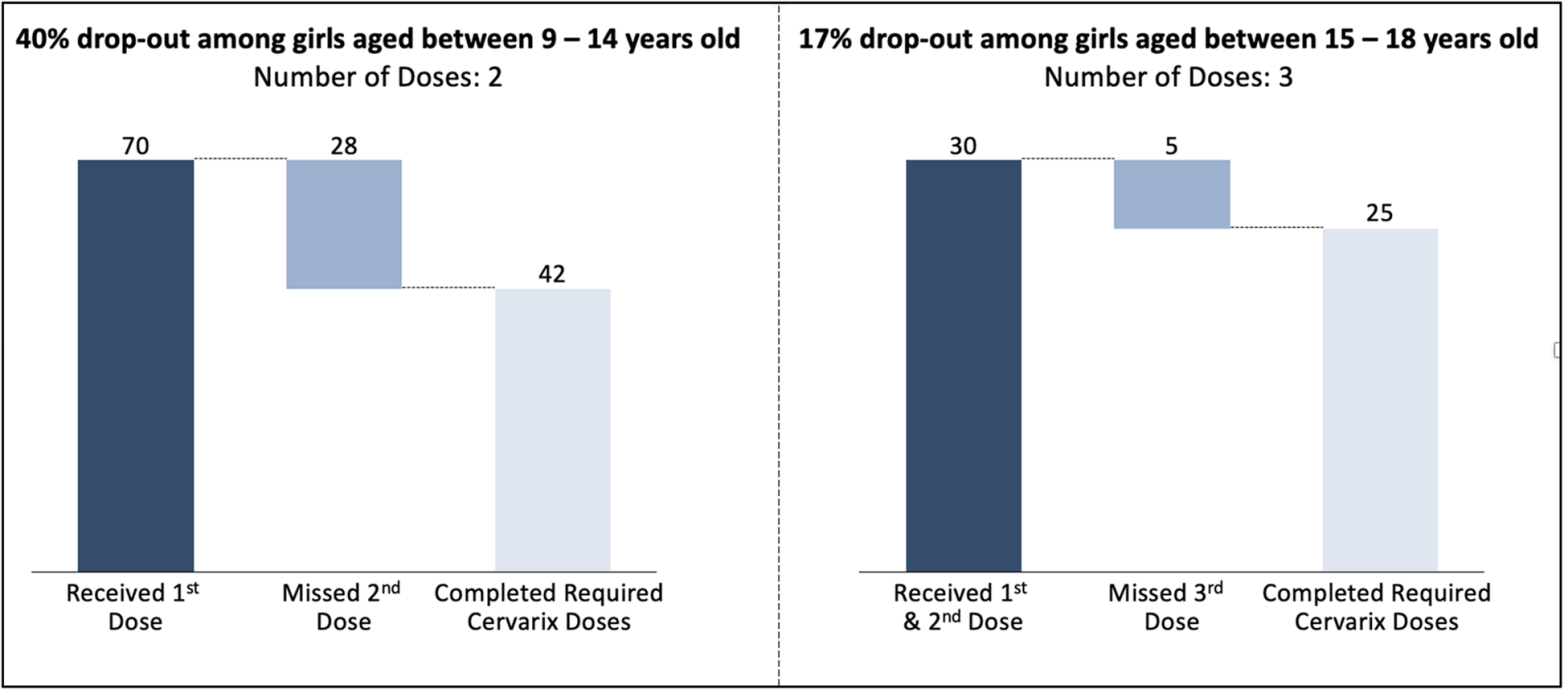
Proportion of girls that did not complete HPV vaccine dosage within 6 months of 1st dose

All participants received the first HPV vaccine dose. Of the 70 girls between the ages of 9–14 who received their 1st dose, 42 completed the required 2nd dose within the 6 months window. For girls between the ages of 15–18, 30 received their 1st dose and 25 completed the required 3 doses within the 6 months window. Twenty-eight junior students (9–14 years) and five senior students (15–18 years) missed the 6-month interval dose. In total, 33 girls could not complete the vaccination within 6 months of their first dose due to school closures mandated because of the COVID-19 pandemic. Complete vaccination of girls under 14 was 60% (42/70) and 83% (25/30) for girls ages 15 and above (Fig. 1).

### Community member perceptions of HPV

Initial perceptions of the vaccine were poor. Many parents in the community believed that HPV vaccine is used for birth control which reduced their wiliness to provide vaccination consent. Additionally, parents were apprehensive of the vaccination program and were concerned the students were being used as “Guinea-pigs” specifically due to their status as a low resource community:

*“Why didn’t you vaccinate children in the urban area whose parents are enlightened to know the consequences of the vaccine, you probably chose us because we are not enlightened and not financially buoyant to sue if any issue arises due to the vaccination” -* ***parent of student.***

## Discussion

HPV vaccine introduction is different to infant vaccination programs as it is targeted at a new user group (9–14-year-old) for which no existing platforms exist. Furthermore, sensitivities around sexual activity have been shown to raise concerns by parents of adolescent girls targeted for the vaccine [23]. The WHO recommends that HPV delivery strategies are targeted to country specific health system and sociocultural contexts [2]. Understanding the factors that influence HPV vaccine uptake such as cultural acceptance and delivery feasibility is crucial for decision-makers. This study provides perspectives on knowledge, attitudes and perceptions of the HPV vaccine from a pilot program among school aged girls within a rural community in Northern Nigeria where research is lacking.

### HPV vaccination knowledge and attitudes

Knowledge about HPV and cervical cancer is fair in adolescent girls in Yauri GSSC. However, as expected, girls in at higher secondary school level had higher knowledge scores compared to those in the lower secondary level classes. A study in India showed that older college students were more aware about HPV and cervical cancer than their younger and male counterparts [24]. This points to the importance of education in awareness about cervical cancer and its prevention by the HPV vaccine.

### Community sensitization

Effective community sensitization prior to vaccine introduction is vital to community acceptability [25–27]. Advocacy plays a major role in shifting stakeholders’ views towards the HPV vaccine [28, 29]. In Yauri, advocacy efforts improved community acceptance of the vaccine. Initial reports of vaccine introduction triggered negative messages in the community; members of the community feared the vaccine was for birth control and withheld support. However, following sensitization and awareness with teachers and health workers, more individuals were receptive to the vaccine. As in other studies, convincing parents on the severity of cervical cancer and the preventative benefits of the vaccine is necessary to obtain their support and consent for vaccination [27].

### Partnership with key influencers and stakeholders

A key component of HPV vaccine delivery is multi-stakeholder collaboration. Several studies show that coordination and collaboration between ministries of health, development partners, the education sector, community-based and professional groups is a key driver of vaccine uptake [8, 9, 30]. Our study largely supports these findings, coordination between teachers, health care workers, and community leaders was crucial to the success of the pilot program in Yauri. Students and community members attributed successful messaging to health workers embedded in the community who deconstructed key health information and clarified questions on the vaccines, and teachers who effectively followed up with students and parents.

Our findings also highlight the importance of selecting appropriate mechanisms and actors for dissemination of key messages. In our study setting, teachers played a key role in obtaining parental consent for the pilot program. A study by Masika et al., suggests that empowering teachers as vaccine champions may be a feasible way to increase awareness and disseminate key messages on the HPV vaccine and cervical cancer [31].

In Yauri, parent groups and community leaders were also identified as key influencers of HPV vaccination. The study had to be defended to the Parents Teachers Association of GSSC Yauri who served as gatekeepers to the school. Additionally, the most important gatekeeper for the entire project was the Emir of Yauri, an eminent traditional and religious leader who provided community approval for the pilot project. As the most respected leader in the community, in the absence of his approval and support, the pilot project would have likely failed. These findings highlight the importance of ensuring that all relevant stakeholders are mapped, consulted, and carried along from inception to conclusion including HPV vaccination strategy design, implementation and evaluation.

### Barriers to implementation

#### Costs

Without Universal Health Coverage, vaccine availability remains a major barrier to access [14, 17]. None of the study participants could afford the discounted vaccine costs which was considered unaffordable by all the parents of the study participants. The prohibitive cost of the vaccine has been shown to negatively impact attitude toward the vaccine and therefore constitutes a main barrier to vaccine uptake in LMICs [32–35]. In Nigeria, the price of a complete dose of the HPV vaccine ranges from US$30 - US$50 and studies suggest that even at the lowest obtainable public sector price of approximately US$36, study participants consider the vaccines unaffordable [32, 33]. Currently, HPV vaccines are not included as part of the free vaccines offered under the National Immunization Programme (NIP) in Nigeria and are paid for out-of-pocket by the recipient [20, 34, 36]. Considering up to 45% of Nigerians live on less than US$ 2 a day, costs will remain a main barrier to vaccine uptake, specifically in poorer communities. As such, governments must prioritize funding mechanisms to ensure access to vaccines particularly in low resource communities.

Additionally, the HPV vaccine is relatively expensive to procure, and operational costs of vaccine delivery can be substantial. Many countries have found that the cost of implementing an HPV vaccination program is significant [2, 36]. While the vaccine was provided for free, operational costs including engagement of healthcare workers for administration and safe transportation logistics (for health workers and cold chain) significantly increased the vaccination program costs and was one reason for limiting the study to 100 participants.

#### Health workforce

With less than 20 health care workers per 100,000 population, Nigeria faces a shortage in availability of qualified health personnel for vaccine delivery; nurses and CHEWs are already overstretched at their health facilities or not available at all in many communities [37, 38]. Health care worker supervision, allowances and transportation costs have frequently been reported as been drivers of high vaccine delivery costs [6]. Such costs need to be considered particularly in rural communities where vaccination campaigns may require health workers from outside the target community, increasing vaccine delivery costs.

#### Infrastructure

The logistics of vaccine delivery from state and LGA cold stores to the school posed a considerable challenge. The established vaccine supply chain in Nigeria currently ends at Primary Health Centers (PHCs), therefore reaching rural, hard to reach areas is hindered by lack of infrastructure which further increases costs on an already underfunded system [39, 40]. For this study, vaccines were stored in a nearby town with cold storage facilities and provisions were made for power using generators further increasing program costs. With ambient temperatures greater than 37 °C, maintaining cold chain was critical. The team had to mitigate for lack of constant electricity supply in Yauri by relying on low technology, portable cold chain containers. Studies in low resource settings show there is a large risk of exposing vaccines to temperatures above the limit of 2^o^ to 8 °C and programs must account for effective cold chain in their planning [41]. Overall, the study points to the careful planning required for vaccine delivery in rural hard-to-reach communities to ensure effective supply chain to the target user.

#### COVID-19

The biggest unforeseen challenge was the COVID-19 pandemic and subsequent school closures which impacted the study. Consequently, the cohort could not be reached in school as intended after the first dose, highlighting the importance of risk assessment and a limitation of the school-based vaccine delivery approach. While this approach is effective and convenient at reaching a large number of the target audience at a central location, it needs to be complemented with a method designed to reach those who are out of school [2, 42, 43]. Leveraging the strong buy-in from the Emir and program support by the community, the project team returned in November 2020 to vaccinate girls that had not completed their dosage within the recommended 6 months schedule. On the follow up vaccination, the 5 girls aged 15 and above were not present and lost to follow up. In Nigeria, 10.5 million children aged 5–14 are not in school [13]. The numbers are even more grim in the Northern states like Kebbi state where almost 50% of girls are not enrolled in primary school [12]. A national school-based HPV vaccination strategy must include an approach that targets out of school children as well as strategies to maximize follow up such as verification of two points of contact for every schoolgirl to minimize drop-out rates and maximize vaccine coverage.

### Study limitations

The study adopted the purposive sampling technique (a non-probability sampling technique) to select GSSC Yauri. Therefore, the findings of this study may not be generalizable to other study settings. Our methods of obtaining parental consent may have introduced selection bias into the study; obtaining parental consent over the phone may have introduced systematic bias by excluding students whose parents did not have phone access. Additionally, we were not able to collect data from students whose parents refused consent. It can be argued that those with parental refusal differ in their knowledge and attitudes towards the vaccine compared to our study participants. Notwithstanding, we believe the results of this study are aligned with findings in similar studies conducted in low resource settings. Therefore, these findings can be useful to other researchers and decision makers working on HPV vaccination delivery.

## Conclusion

The present study underscores the importance of designing HPV campaigns tailored to context specific realities. Despite the challenges of vaccine delivery in low resource settings, HPV vaccines can be successfully provided to girls in hard-to-reach areas. While participants lacked specific knowledge about HPV infection and cervical cancer, their attitude towards receiving the vaccine following sensitization was positive. However, decision-makers must consider financing mechanisms to ensure vaccine access, as the vaccine was considered unaffordable. Vaccine delivery costs to hard-to-reach communities are further exacerbated by health system limitations including health worker availability, infrastructure and logistics. Social awareness and mobilization anchored by community influencers is essential to improving acceptance and therefore vaccination uptake. Overall, the success of the vaccination program in Yauri, Kebbi is attributable to careful program planning to overcome health system limitations, advocacy, and community buy-in facilitated by key community influencers.

## Data Availability

The datasets used and/or analyzed during the current study available from the corresponding author on reasonable request.
